# Contributions of *PHOX2B* in the Pathogenesis of Hirschsprung Disease

**DOI:** 10.1371/journal.pone.0054043

**Published:** 2013-01-14

**Authors:** Raquel María Fernández, Yves Mathieu, Berta Luzón-Toro, Rocío Núñez-Torres, Antonio González-Meneses, Guillermo Antiñolo, Jeanne Amiel, Salud Borrego

**Affiliations:** 1 Department of Genetics, Reproduction and Fetal Medicine, Institute of Biomedicine of Seville, University Hospital Virgen del Rocío/Centro Superior de Investigaciones Científicas/University of Seville, Seville, Spain; 2 Centre for Biomedical Network Research on Rare Diseases, Seville, Spain; 3 INSERM U-781, AP-HP Hôpital Necker-Enfants Malades, Paris, France; 4 Department of Paediatrics. University Hospital Virgen del Rocío, Seville, Spain; Institut Jacques Monod, France

## Abstract

Hirschsprung disease (HSCR) is a congenital malformation of the hindgut resulting from a disruption of neural crest cell migration during embryonic development. It has a complex genetic aetiology with several genes involved in its pathogenesis. *PHOX2B* plays a key function in the development of neural crest derivatives, and heterozygous mutations cause a complex dysautonomia associating HSCR, Congenital Central Hypoventilation Syndrome (CCHS) and neuroblastoma (NB) in various combinations. In order to determine the role of *PHOX2B* in isolated HSCR, we performed a mutational screening in a cohort of 207 Spanish HSCR patients. Our most relevant finding has been the identification of a *de novo* and novel deletion (c.393_410del18) in a patient with HSCR. Results of *in silico* and functional assays support its pathogenic effect related to HSCR. Therefore our results support that PHOX2B loss-of-function is a rare cause of HSCR phenotype.

## Introduction

The paired-like homeobox 2b gene (*PHOX2B*) encodes a transcription factor involved in the development of several noradrenergic neuron populations in mice. In the murine model, *Phox2b* expression starts as soon as enteroblasts invade the foregut mesenchyme and is maintained throughout the development into enteric neurons, so that homozygous disruption of *Phox2b* in mice leads to an absence of enteric ganglia [1]. *PHOX2B* is the major disease causing gene in Congenital Central Hypoventilation Syndrome (CCHS, OMIM 209880), that is associated with sympathetic tumour and Hirschsprung disease (HSCR, OMIM 142623) in 5 and 20% of cases respectively. *PHOX2B* mutations are mainly alanine expansions within the 20-residue polyalanine tract and less frequently frameshift, missense and nonsense mutations, and by partial or whole gene deletions [2]–[7]. In some cases *PHOX2B* point mutations/deletions have been reported in patients without CCHS and presenting neuroblastoma (NB, OMIM 256700), HSCR or both [6], [8], [9]. Specifically, only two cases of HSCR not associated to either CCHS or NB have been reported so far. The first one was a girl who presented with a syndromic short segment HSCR associated with a *de novo* t(4;8)(p13;p22) translocation. A comparative genomic hybridization (CGH) study found a 4p12p13 deletion that included *PHOX2B* [8]. The second case correspond to several members of one family harboring the p.Arg100Leu point mutation, that resulted in either isolated HSCR, ganglioneuroma or NB [9]. Taking all these data together, *PHOX2B* can be also regarded as an interesting candidate gene to be further studied in isolated forms of HSCR. In this sense, an association of the common *PHOX2B* g.1364A>G polymorphism has been reported for the Chinese HSCR series [10], although whether it directly contributes to disease susceptibility or represents a marker for a locus in LD with *PHOX2B* in such population needs further investigation. HSCR is characterized by the absence of intramural ganglion cells in the myenteric and submucosal plexuses along a variable portion of the distal gastrointestinal tract, which resembles the phenotype presented by the Phox2b knockout mice [1]. HSCR is a model in the study of diseases with a complex mode of inheritance. When isolated, the transmission is usually non-Mendelian with low, sex dependent penetrance. A multiplicative model with several loci involved is the current most accepted model for the genetic basis of HSCR, with the *RET* proto-oncogene being the major disease causing gene. Indeed, almost all isolated HSCR patients harbour either a heterozygous mutation of the coding sequence or, more often, a hypomorphic allele located in a conserved sequence in intron 1 and acting as a transcriptional enhancer [11], [12].

In the present study we have sought to determine if *PHOX2B* plays a role in the pathogenesis of HSCR phenotype, by a mutational screening of its coding sequence in a series of 207 Spanish HSCR patients.

## Materials and Methods

### Patients and Control Subjects

A total of 207 HSCR patients from Spain have been included in this study (23% female, 77% male) and their parents when available. 185 of these patients were sporadic cases, whereas 23 were familial cases belonging to 12 different families. In addition, we have also included a group of 150 normal controls comprising unselected, unrelated, race, age, and sex-matched individuals. An informed consent was obtained from all the participants for clinical and molecular genetic studies. The study conformed to the tenets of the declaration of Helsinki as well as the requirements established by our Institutional Review Board.

### Mutational Screening of *PHOX2B*

Mutational screening of *PHOX2B* coding sequence was undertaken by denaturing high-performance liquid chromatography (dHPLC). Primers used for PCR amplification and annealing temperatures are summarized in Table 1. The dHPLC analyses were carried out on an automated HPLC device equipped with a DNA separation column using the WAVE DNA Fragment Analysis system (Transgenomic). PCR products were denatured for 3 min at 95°C and then gradually re-annealed by decreasing sample temperature from 95°C to 65°C over a period of 30 min. Five µL of PCR products were then injected onto the DNASeq cartridge and eluted at a constant flow rate of 0.9 ml/min through a linear acetonitrile gradient. DNA fragments were detected by the system’s UV detector and analyzed as chromatograms. Oven temperature for optimal heteroduplex separation under partial DNA denaturation was determined for each amplicon using the WAVEMarker software (Table 1). Those samples with aberrant wave profiles were subjected to sequence analysis by direct sequencing with a ABI 3730 DNA Analyzer (Applied Biosystems). The alignment of our results with the sequences provided for *PHOX2B* (RefSeq NM_003924.3) was carried out using the software SeqScape v.2.5.

**Table 1:** Amplification and dHPLC conditions for *PHOX2B* genomic sequence analysis.

			PCR Chemistry						PCR Conditions						
Exon	Primers sequence	Bp	B	CM	DNTPS	P	Taq	DNA	D	DT	H	HT	E	ET	TdHPLC
**1A**	F: TTGGATCAGGGAGAATCGTC; R:AAAAGGTTCTGGATGGCTCA	475	1X	3 mM	0.2 mM	0.05 µM	1 U	10 ng	95°	30′′	61°	30′′	72°	30′′	58.5°
**1B**	F: CTCCAGCCACCTTCTCCATA; R: AATTACCCCTCCCTGCAATC	480	1X	3 mM	0.2 mM	0.05 µM	1 U	10 ng	95°	30′′	57°	30′′	72°	30′′	55°/61.5°
**2**	F: CTGCCGTATGACCTGACCTT; R: CCAGGACTTCGAATTTCACC	377	1X	3 mM	0.2 mM	0.05 µM	1 U	10 ng	95°	30′′	57°	30′′	72°	30′′	61°/64.2°
**3**	F: ACCGTCTCTCCTTCCGTCTT;R: ACAATAGCCTTGGGCCTACC	686	5 µL	1 mM	0.2 mM	0.5 µM	0.5 U	25 ng	95°	1′	57°	1′	72°	45′′	62.6°/64.5°

Bp: size of the fragment; B: buffer concentration; CM: MgCl2 concentration; DNTPs: dNTPs concentration; P: primers concentration; Taq: units of Taq polymerase; DNA: DNA quantity; D: Denaturing temperature; DT: Denaturing time; H: Hybridization temperature; HT: Hybridization time; E: Extension temperature; ET: Extension time; T dHPLC: Injection temperature in dHPLC. The general PCR protocol was [95°C-5′]→[(D–DT) →(H–HT) → (E–ET)]×35cycles→[72°C–7′]→4°C.

### Bioinformatic Analysis

For evaluation of the inter-species conservation of the residues we used the CLUSTAL tool (http://www.ebi.ac.uk/Tools/clustalw2/index.html). Functional domains of PHOX2B protein was predicted with ScanProSite (http://expasy.org/tools/scanprosite/). Prediction of secondary structure of protein was performed with GARNIER software (http://emboss.bioinformatics.nl/cgi-bin/emboss/garnier).

### Splicing Analysis

PCR products containing either the Wild type (WT) or the c.393_410del18 (del18) mutation were amplified using primers containining both of them an artificial site for Kpn1 (sequences can be asked on requests) and as template a DNA sample of the patient heterozygous for the mutation. PCR amplicons of 401 bp containing the entire exon 2 and exon-intron boundaries of Phox2b (188 bp) starting 112 bp upstream and finishing 101 bp downstream of Phox2b exon2 respectively. Amplicons were then digested with Asp718 (*iso*-schizomer of KpnI) for convenient reasons to subclone it into the Exontrap cloning system (MoBiTec) at the multiple cloning site previously digested with Asp718 and dephosphorylated using antartic phosphatase (New England Biolabs inc.) before ligation with the T4 DNA ligase (New England Biolab inc.). Constructs were transformed into competent bacteria and grown onto ampicilline agar plate to generate bacteria clones containing either the WT or the del18 construct. Ten clones were picked randomly and inoculated for amplification of the constructs. Midiprep Purified (Macherey-Nagel) constructs were then verified by sequencing. HeLa cells were grown to 80% confluency in Dulbecco’s minimum essential medium (DMEM) supplemented with 10% fetal bovine serum in 6-well plates. Constructs containing an insert (WT or del18) were then transfected respectively into Hela cells using Fugene HD (Roche) as transfectant (1g of plasmid/well). After 2 days of culture, cells were harvested, their RNA was isolated (Qiagen kit) and RT- PCR using the 5′ and 3′ primers of the Exontrap cloning system followed by bidirectional sequencing using the same primers was performed to reveal the splicing outcome.

### Luciferase Assay

Human PHOX2B cDNA was obtained from MRC Geneservice (CLONE ID 3161284) in a pOTB7 vector. The PHOX2B cDNA insert was isolated from pOTB7 by EcoRI-BglII digestion and introduced in the pcDNA 3.1/zeo (−) expression vector (Invitrogen) at EcoRI-BamHI sites. The c.393_410del18bp mutation was generated using the Phusion Site-Directed Mutagenesis Kit (Finnzymes) according to the manufacturer’s protocol using forward and reverse primers phosphorylated at the 5′ end and the GC-RICH PCR system buffer (Roche) instead of the HT buffer (Finnzymes). All constructs were validated by DNA sequencing. The DBH luciferase reporter construct was previously described [6], [13]. Plasmids used for luciferase assays were prepared using Qiagen kit. HeLa cells were grown to 80% confluency in Dulbecco’s minimum essential medium (DMEM) supplemented with 10% fetal bovine serum in 6-well plates. Cells were transfected with 900 ng of recombinant pcDNA 3.1/zeo-PHOX2B expression vector, 1.2 µg of the firefly luciferase reporter promoter, 30 ng of pRL-CMV Renilla luciferase internal control (Promega), and 4 µl of Fugene HD (Roche) in 100 µl of OPTI-MEM (Invitrogen). Cells were harvested and lysed 24 h to 48 h after transfection. Firefly and Renilla luciferase activities were assayed according to the manufacturer’s protocol (Dual-luciferase reporter assay system, Promega) and normalized by the internal control pRL-CMV. Experiments were repeated 3 times in duplicate.

## Results

We have detected a total of 5 novel *PHOX2B* variants in the mutational screening of the 207 HSCR patients, consisting in 2 heterozygous nucleotidic variants and 3 heterozygous indels (Table 2). Nucleotidic variations (c.354G>C, p.Ala118Ala and c.242-76T>C) seem to be non pathogenic variants because of their nature, their presence in control population and the benign effect predicted by the bioinformatic tools (http://www.fruitfly.org/cgi-bin/seq_tools/splice.pl). Regarding the indels, 2 of them (c.741_761del21bp, found in two patients, and c.745_765del21bp) were located in the polyalanine region of exon 3 encompassing 21 bp and resulted in the deletion of 7 alanines. Both of them are also considered as non-pathogenic polymorphisms [14].

**Table 2:** *PHOX2B* variants identified in HSCR patients.

Variant	Sporadic/Familial^a^	Parent origin of the mutation	Presence in control population	HSCR Phenotype
c.242-76T>C	Sporadic	Mother	Yes	S-HSCR
c.354G>C	Sporadic	*De novo*	No	L-HSCR
c.393_410del18bp	Sporadic	De novo	No	L-HSCR
c.741_761del21bp	Sporadic	Mother	No	L-HSCR
c.741_761del21bp	Familial	Mother	No	L-HSCR
c.745_765del21bp	Familial	Mother	No	NA

NA, not available; S-HSCR, short-segment; L-HSCR, long-segment.

a The term “familial” is applied when more than one family member has Hirschsprung disease. Otherwise, when there is no family history, the cases are considered sporadic. The only pathogenic variant is shaded in grey while the remaining novel variations are considered as non-pathogenic.

**Figure 1:**
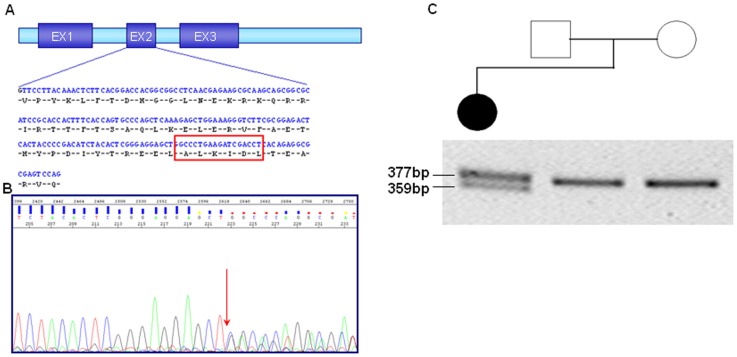
*PHOX2B* deletion detected in exon 2. (A) Schematic representation of *PHOX2B* showing both nucleotide and amino acid sequence of exon 2; the 18bp corresponding to the deletion are boxed. (B) Sequence chromatogram of *PHOX2B* exon 2; the arrow indicates the beginning of c.393_410del18bp. (C) Pedigree and agarose gel electrophoresis of the amplification product of exon 2 in the patient and her parents.

More interestingly, in a sporadic female patient we identified a heterozygous in-frame deletion of 18 nucleotides in the exon 2 of *PHOX2B*, located between nucleotides 393 and 411, and resulting in the lost of 6 aminoacids (Ala-Leu-Lys-Ile-Asp-Leu) within the homeodomain of *PHOX2B* (c.393_410del18bp, p.(Ala131_Leu136del), Figure 1). Of note, the deletion was absent in 150 control individuals and occurred *de novo* in the patient. The patient is the third girl born from healthy non-consanguineous parents. She was born at term with a birth weight of 3,280 g. Early after birth, a severe constipation and abdominal distension was noticed, Hirschprung disease was diagnosed histologically on intestinal biopsy and aganglionosis extended up to the ascending colon. Surgery using Soave-Boley procedure was performed during her first week of life. Diarrhea due to lactose intolerance was diagnosed at four years old. In early infancy, motor delay and dysmorphic facial features were detected with trigonocephaly and hypertelorism. She also presented speech difficulties that improved with age. She is currently ten years old, and suffers from scoliosis treated by orthopaedic corset and walking difficulties due to genu varum. Her intelligence is in the normal range, she presents strabismus, and no clinical manifestations suggestive of CCHS or NB.

Previous mutational studies showed that this patient also presents the mutation p.Arg93Trp in the *GDNF* gene, inherited from her unaffected mother, as well as the *RET* enhancer variant (c.73+9277T, rs2435357) in homozygous state. No other mutations were identified in the other HSCR genes analyzed (*RET, NRTN, PSPN, ARTN, EDNRB, EDN3, SOX10, PROK1, PROKR1, PROKR2, NRG1, SEMA3A* and *SEMA3D*).

**Figure 2:**
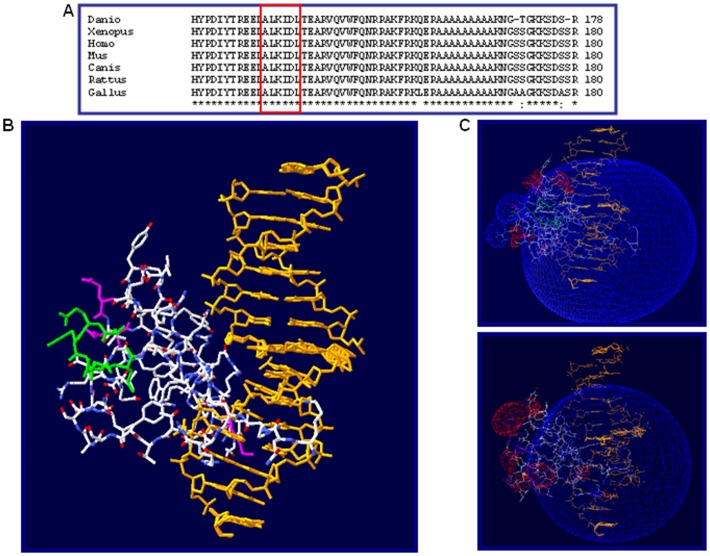
Structure of the homeodomain. (A) Multiple sequence alignment among different species. Aminoacid residues involved in the deletion are included within a red box. (B) Three-dimensional representation of the interaction between the *Drosophila sp.* homeodomain and the target DNA sequence (orange). Aminoacids encompassed by the deletion are represented in green. C) Prediction of the electrostatic potential for the wild type protein (up) and the mutated one (down). Negative charge is represented in blue and positive charge in red.

In silico, the 6 deleted amino acids are located within a highly conserved region within the homeodomain of the protein and with a significant structural role (CLUSTAL, ScanProSite and GARNIER software) being next to a Valine and an Asparagine, essential for DNA recognition, as well as to a Glutamic acid and an Arginine implicated in the water-mediated hydrogen bond formation of the recognition helices of the two homeodomains (Figure 2) [15]. Crystal structure of this functional domain, the homeodomain, had been solved in *Drosophila sp*. by Wilson and Desplan in 1995 [15]. Mutational studies on this structure (PDB ascession code 1FJL) using Swiss-Pdb Viewer software v 3.7 shows that spatial and charge distribution could be altered with respect to wild type homeodomain (Figure 2).

**Figure 3:**
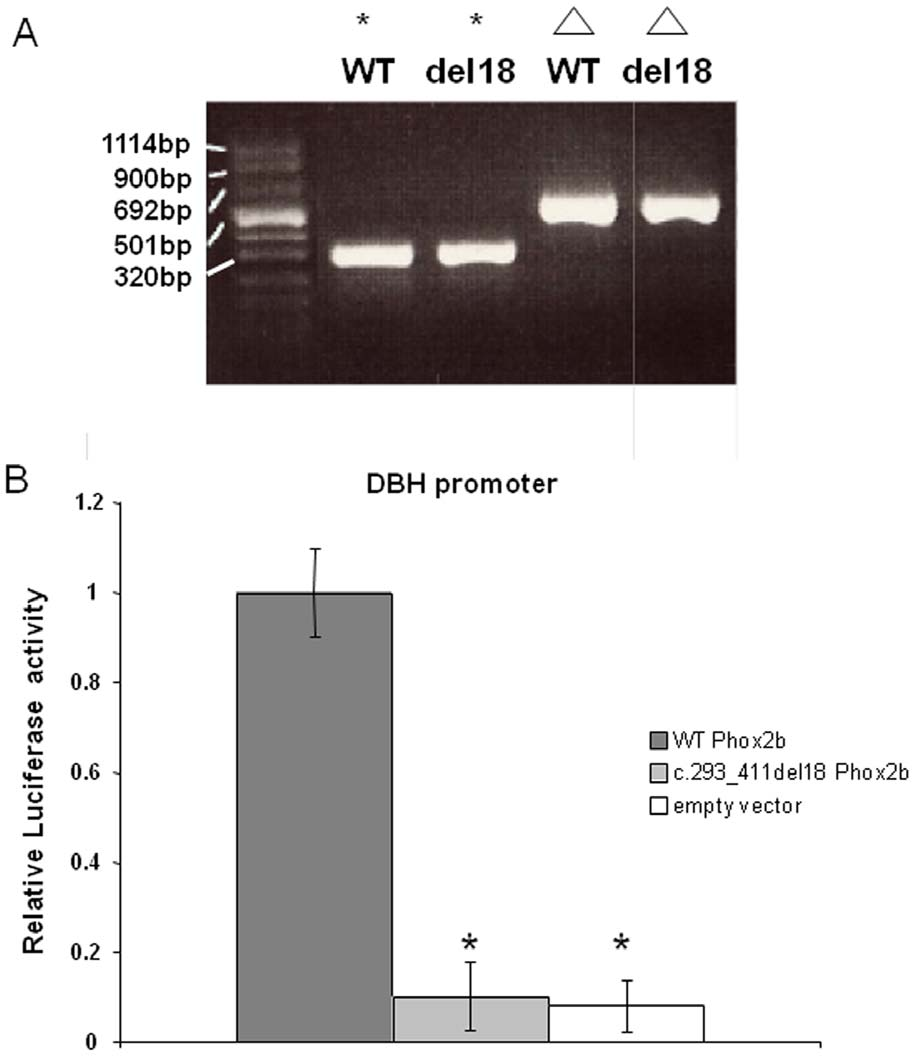
Functional assays on the *PHOX2B* c.393_410del18 mutation (A) Reverse transcribed PCR analysis of Hela cells transfected with the respective Exontrap wild type and mutated exon 2 constructs. Wild type and the 393_410del18 mutated inserted human Phox2b sequences into Exontrap vectors in the correct 5′->3′ orientation have spliced their exon 2 (indicated by a triangle) as expected (approx. 500 bp, 5′ and 3′ exons of Exontrap+Phox2b exon2) demonstrating that the c.393_410del18 mutation does not seem to affect the splicing of the exon2 of Phox2b. In contrast when the exon 2 human Phox2b sequence is inverted 3′->5′ (indicated by an asterisk) within the Exontrap construct for the wild type as for the mutated sequence, the entire Phox2b insert+Exontrap-introns sequences are spliced out (approx. 300 bp). (B) Transactivation of the DBH promoter. The error bars indicate SD for n = 3 duplicate experiments. Each luciferase activity has been normalized with an internal control (pRL-CMV, Promega). In each experiment, the activity of the wild type protein was set to one, and the results are shown as the ratio between the activities of the mutant and the wild-type proteins. Significant differences between the wild-type and the specific mutant are indicated by an asterisk (Student’s test, *p<0.01).

Subsequently we further performed both a splicing analysis and a luciferase assay to verify the functional effect of the mutation. There is an increasing number of studies showing that mutations within both exons and introns, but outside of the canonical splice sites, can also disrupt splicing [16]. In this sense, exonic variants may result in deleterious effects by activating a *de novo* ectopic splice site, which is then used in preference to the natural splice site, shortening the exon. Then, we decided to test if the new mutation affected the splicing process, but this analysis demonstrated that Phox2b exon 2 insert containing the del18 mutation had been spliced as its corresponding WT insert sequence (Figure 3A), and as in human Phox2b mRNA transcripts (same size of RT-PCR product). Therefore we subsequently assessed the ability of the c.393_410del18 PHOX2B mutant described in Figure 1 to transactivate the dopamine-β-hydroxylase (DβH) promoter. Transactivation activity of the mutant proteins was almost, if not completely, abolished compared to the WT (Figure 3B). A western blot of the total extract from the luciferase assay shows that the mutant protein is produced in comparable amounts than the WT protein (data not shown). Thus we conclude that del18 leads to a null allele.

## Discussion

*PHOX2B* has been described as the major locus associated with CCHS and the first NB predisposing gene identified. However, except for 2 whole deletions of *PHOX2B* gene due to a large chromosomal rearrangements and 1 point mutation, *PHOX2B* mutations have been seldom reported in cases with HSCR phenotype not associated to CCHS or NB [7]–[11], [17].

The most relevant finding in the mutational screening is one deletion involving the lost of 6 amino acids within the functional domain of *PHOX2B*. These residues are located in a high conserved region of the homeodomain and for this reason we postulate that their deletion could modify the PHOX2B transactivation ability, in the same way that it has been proven for other missense mutations by in vitro approaches [18]. In addition, bioinformatic tools predict a charge distribution variation that supports our hypothesis. Briefly, alterations of the high conserved homeodomain seem to entail totally the protein function, as the luciferase assay demonstrates through the abolishment of the transactivation of the DβH promoter.

As it has been described, PHOX2B is a key factor involved in the transcriptional regulation of *RET* [1], the major gene for HSCR. However, no putative PHOX2B binding site has been identified in *RET* promoter implying that PHOX2B may act as a bridge to recruit other transcriptional regulators, such as, Hes1 and KLF4, to mediate *RET* transcription [19]. It has been suggested that central ventilatory neurons may be more sensitive to *PHOX2B* mutations that affect protein misfolding, oligomerisation or gain-of-function mutations (ie alanine expansion), whereas enteric phenotype might be associated to mutations related with gene dosage or missense mutations which retains some transcriptional activity but do not present oligomerisation properties, as would be the case of the mutation here reported [6], [8], [18]. With this case, the hypothesis of PHOX2B loss of function of 50% damage for a correct ENS development is reinforced. Together with the PHOX2B mutation, our patient also presents the enhancer RET variant (c.73+9277T, rs2435357) in homozygous state, which has been reported to compromise RET transactivation by SOX10 [12]. Of note, it had been reported that such frequent, low penetrant, predisposing allele of the RET gene can be regarded as a risk factor for the HSCR phenotype in CCHS syndrome [20]. Moreover, the patient also carries the p.Arg93Trp mutation in the *GDNF* gene, inherited from her unaffected mother. These findings would be concordant with an additive/multiplicative model in which those mutational events could be acting exacerbating the phenotype, as it has been previously proposed [21]–[23]. In summary, this finding constitutes a new perspective of the *PHOX2B* role in the pathogenesis of HSCR. Further studies in additional series of patients are necessary to deepen in our knowledge of the association between Hirschsprung disease and *PHOX2B* gene.
